# Application of Five Different *Chlorella* sp. Microalgal Strains for the Treatment of Vegetation Waters Derived from Unconventional Oil Extractions Enriched with Citrus Byproducts

**DOI:** 10.3390/foods11101398

**Published:** 2022-05-12

**Authors:** Monica Macaluso, Carolina Chiellini, Adriana Ciurli, Lorenzo Guglielminetti, Basma Najar, Isabella Taglieri, Chiara Sanmartin, Alessandro Bianchi, Francesca Venturi, Angela Zinnai

**Affiliations:** 1 Department of Agriculture, Food and Environment, University of Pisa, Via del Borghetto 80, 54126 Pisa, Italy; monica.macaluso@phd.unipi.it (M.M.); adriana.ciurli@unipi.it (A.C.); lorenzo.guglielminetti@unipi.it (L.G.); basma.najar@agr.unipi.it (B.N.); isabella.taglieri@unipi.it (I.T.); chiara.sanmartin@unipi.it (C.S.); alessandro.bianchi@phd.unipi.it (A.B.); francesca.venturi@unipi.it (F.V.); angela.zinnai@unipi.it (A.Z.); 2 Italian National Research Council, Institute of Agricultural Biology and Biotechnology, Via Moruzzi 1, 56124 Pisa, Italy; 3 Interdepartmental Research Centre “Nutraceuticals and Food for Health”, University of Pisa, Via del Borghetto 80, 56124 Pisa, Italy

**Keywords:** olive-oil mill wastewater, wastewater, microalgae, *Chlorella* sp., phenolic compounds

## Abstract

The Mediterranean diet has, among its cornerstones, the use of olive oil for its nutraceutical and organoleptic properties. Despite the numerous merits, olive-oil mill wastewater (OMWW), which is generated by the olive-oil extraction process, is one of the most serious environmental pollutants in the Mediterranean countries. The polluting potential of OMWW is due to its high content of tannins, polyphenols, polyalcohols, pectins and lipids. In order to close the recovery cycle of a fortified citrus olive oils previously developed, we tested the ability of five microalgae of the Chlorella group (SEC_LI_ChL_1, CL_Sc, CL_Ch, FB and Idr) in lowering the percentage of total phenolic compounds in vegetation water. This was obtained with three different extraction processes (conventional, and lemon and orange peels) at three concentrations each (10%, 25% and 50%). The results showed that strains Idr, FB and CL_Sc from the Lake Massaciuccoli can tolerate vegetation water from conventional and lemon peel extractions up to 25%; these strains can also reduce the phenolic compounds within the tests. The application of microalgae for OMWW treatment represents an interesting opportunity as well as an eco-friendly low-cost solution to be developed within companies as a full-scale approach, which could be applied to obtain a fortified microalgal biomass to be employed in nutraceutical fields.

## 1. Introduction

One of the biggest problems in olive-oil production is the enormous amount of solid and liquid waste produced by the extraction process, called olive-oil mill wastewater (OMWW). The high phenolic nature of OMWW and its organic contents make it highly resistant to biodegradation. Their composition is variable depending on a wide range of influences such as climate, cultivation and the particular milling method used in oil extraction [1,2].

According to Bettazzi and coworkers, the chemical oxygen demand (COD) of OMWW samples can range from 35 to 200 g/L, the biochemical oxygen demand (BOD) can range from 15 to 135 g/L, suspended solids (SS) can range from 6 to 69 g/L, total phenols can range from 2 to 15 g/L, while pH ranges can range from 4.5 to 5.8 [3]. This is one of the highest organic loads of all known concentrated effluents, and it is 100–200 times higher than domestic wastewater.

A number of processes have been previously used for OMWW treatment, including lagooning, physio-chemical treatment, electro coagulation, Fenton and electro Fenton processes [1]. Chemical treatment is the most common for OMWW with membrane separation [4]. However, some reports found significant disadvantages in these treatment methods and showed that no single technology can treat OMWW effectively as a stand-alone process [1].

In recent decades, the increasing need to find new, alternative, eco-friendly and economically sustainable solutions to the common water treatments resulted in the development of biological strategies, which are mainly based on the use of microbial biomasses that help almost completely remove nitrogen [5], sulfur [6], and phosphate [7] as well as decrease BOD and COD (biological/chemical oxygen demand). Microbial biomasses commonly used in wastewater (WW) treatment are mainly composed by bacteria, but a central role is also played by fungal biomasses [8], protists [9], and microalgae [10]. It has been widely demonstrated that microalgae can find an optimal growth medium both in domestic [11] and industrial [12] WW because they require high amounts of nitrogen and phosphorous for their growth as well as organic matter and carbon sources that are all present in large quantities in WWs [13]. Interestingly, microalgae in wastewater treatment can be applied both as living or dead biomass [10]. Microalgae are able to significantly decrease the amount of nutrients and, thus, to reduce BOD [14]. In this context, the application of microalgae could represent a useful alternative in processing methods for OMWW treatment and represents a possible solution for developing new agro-food chains derived by food waste.

In the perspective of food supply chain sustainability, in a previous work [15], waste from the citrus fruit supply chain (orange and lemon peels) was used in order to obtain fortified citrus olive oils with enhanced nutritional properties through a process of cryomaceration and subsequent co-extraction of citrus peels with olives. However, the oils produced, despite the inestimable qualities and virtues conferred by the bioactive compounds of citrus fruits, create production waste that is difficult to dispose of, as they are particularly rich in bioactive compounds. In this context, the first purpose of this work was therefore to close the recovery cycle of the citrus-flavored olive oils, promoting the removal of the originated OMWW; the second was to test whether microalgae could be putative candidates for the treatment of vegetation water and whether they were able to reduce the level of phenolic compounds from the matrix. The vegetation water obtained with three different extraction processes [15] were evaluated (conventional, and lemon and orange peels) at three concentrations each (10%, 25% and 50%). Five environmental microalgal strains belonging to the *Chlorella* group were tested in a 7-day experiment in the presence of different crude vegetation waters and under different light conditions. The quantification of photosynthetic pigments was considered a growth parameter for the microalgal strains exposed to the different treatments [16] compared with the control test (the growth in the absence of vegetation water, indicated as 0%). At the end of the experiment, the reduction in the phenolic compounds was evaluated.

## 2. Materials and Methods

### 2.1. Microalgal Strains and Growth Conditions

The five microalgal strains selected for this experiment were of environmental origin. Strain SEC_Li_ChL_1 was sampled in an artificial lake in Rosignano Marittimo (LI), Italy (43°27′45.34″ N, 10°28′24.42″ E), and was characterized as belonging to the *Chlorella*-Micractinium clade [17]. Strains Idr, CL_Sc, CL_Ch and FB were sampled from the Lake Massaciuccoli (43°50′00.0″ N, 10°19′59.9960″ E) in different sites of the lake area; they were all characterized as belonging to the *Chlorella sorokiniana* group [18]. All five strains were grown in Tris-Ammonium Phosphate (TAP) medium [19] and maintained in the laboratory collection of the Department of Agricultural, Food and Agro-environmental Sciences under the growth conditions described in Chiellini et al. [16].

### 2.2. Citrus Olive Oil Extraction and Wastewater Sample

Orange and lemon citrus peels (came from Massa) were cryomacerated with solid carbon dioxide (1:1 in weight) overnight and then directly added (22% in weight) to olives before milling. The extraction was carried out using a micro oil mill (Oliomio Baby^®^, by “Toscana Enologica Mori”, Tavernelle Val Di Pesa, Florence, Italy) able to mill 20–30 kg of olives. The technical characteristics of the micro-oil mill and the working conditions used followed the method previously described [15].

After the olive-oil extraction, the wastewater was separated from the olive pomace with a laboratory centrifugation treatment (4000 rpm × 5 min). All of the obtained samples were stored at 4 °C under nitrogen for 24 h before the microalgae treatment.

### 2.3. Experimental Setup

Abided by Chiellini’s method [16], the strains were grown in sterile glass tubes in a total volume of 20 mL under a light condition of 16/08 h day–night cycle with a PPFD of 120 mmol photons m^−1^ s^−1^ from cool-white light lamps (Gavita Lep 330 Plasma fixtures, Gavita Holland Light Emitting Plasma, Gavita International b.v., Rozenburg, Netherlands) and a constant 22/24 °C temperature. In all of the experiments conducted, the microalgal biomass of the initial inoculum was 150 mg ± 0.2 mg of fresh weight for each strain in each tube. Three dilutions (in TAP medium) were tested for each kind of vegetation water—10%, 25% and 50%—while a 100% TAP medium was used as control hereinafter, indicated as the “Ctrl_0%” test. In a first experiment, all five strains (SEC_Li_ChL_1, Idr, CL_Sc, CL_Ch and FB) were tested against the crude vegetation water without any preliminary treatment. Three different vegetation waters were tested: (i) conventional, (ii) lemon peel and (iii) orange peel olive-oil extractions.

In the second experiment, we focused our attention on three strains that showed not only growth in the presence of the different vegetation water treatments but also high levels of phenolic compounds removal in the first screening (see the next paragraph for details); these strains were Idr, CL_Sc and FB and were tested against pre-treated vegetation water of conventional and lemon peel olive-oil extraction. The pre-treatment consisted of the recovery of the supernatant obtained after centrifugation (800 rpm, 5 min).

The vegetation water obtained from orange peels was removed from the second experimentation since, according to the previous test, most of the strains did not survive in its presence and most of those that survived were not able to significantly reduce the total phenolic compounds. At the same time, we tested the vegetation water without the microalgae inoculation in the same temperature, oxygen and light conditions to verify the role of the microalgae on decontamination. Both experiments were monitored for one week, and all experiments were performed in triplicate.

### 2.4. Growth Parameters and Total Phenolic Compound Reduction Measure

The microalgal growth parameters measured at the end of the experiment were chlorophyll a, chlorophyll b and total chlorophyll content. The photosynthetic pigment extraction was performed in 100% acetone using the procedure described in Chiellini et al. [16]. Briefly, 1 mL of each sample test was centrifuged (1500 rpm, 5 min at 4 °C), and the pellet was resuspended in 1 mL of 100% acetone (Merk Life Science S.r.l., Milano, Italy) and submitted to 10 min of sonication (Branson 1210, Bransonic Ultrasonic Cleaner, Branson Ultrasonics Corporation, Danbury, CT, USA). The samples were then kept in the dark at 4 °C overnight and subsequently centrifuged (12,000 rpm, 5 min). All of the centrifuges were performed in a Speedmaster 14R, Euro Clone, Milano (Italy). Finally, the absorbance of the supernatant was spectrophotometrically analyzed (UV-1800 Spectrophotometer, Shimadzu, Japan) with regard to the blank at 661.6 and 644.8 nm, according to the equations indicated in Lichtenthaler [20]. The carotenoid content was not measured since the emission spectra of the three different vegetation waters interfered with the wavelength of the carotenoids (470 nm, Supplementary Figure S1a–c for the control, lemon and orange, respectively). The ANOVA (analysis of variance) followed by a pairwise post hoc test was performed using the PAST software [21] on the replicated tests for each treatment.

### 2.5. Analysis of the Phenolic Content

Phenolic substances were determined by Folin–Ciocalteu method, as previously described by Flori [15]. The determination of the total phenols content was expressed in g/L of gallic acid. The phenolic reduction was calculated following Equation (1):

(1) $$\%\;reduction=\left(\frac{C_{0}-C_{f}}{C_{0}}\right)\times 100$$

where ***C_0_*** and ***C_f_*** are, respectively, the concentration of phenols at the beginning and at the end of the experiment. 

### 2.6. HPLC Analysis

#### 2.6.1. Experimental Materials

Acetonitrile (HPLC, Carlo Erba, Carlo Erba Reagenti SpA, Strada Rivoltana km6/7, I-20090 Rodano (MI)), acetic acid (HPLC, Carlo Erba, Carlo Erba Reagenti SpA, Strada Rivoltana km6/7, I-20090 Rodano (MI)) and bi-distilled water (VWR) were used as solvents and mobile phases for HPLC-UV analyses. Six phenolic standards, provided by Extrasynthese, were chosen for the analyses: thyrosol, hydroxytyrosol, 2-cumaric acid, gallic acid, 4-cumaric acid and syringic acid.

#### 2.6.2. HPLC Analysis of Phenolic Compounds

The analysis of phenolic compounds was performed as follows: The sample was first centrifugated for 5 min at 4000 RPM/min, and the supernatant was recovered. The supernatant was then filtered with a Whatman filter (0.22 μm) to then be directly injected into a reversed-Phase C18-X (Adamas^®^ C18-X-Bond 5µ—250 mm × 4.6 mm ID) column of HPLC, which is a system consisting of a PU-2089 Plus quaternary pump (Jasco International Co., Ltd., Tokyo, Japan) equipped with a degasser, an AS-2057 Plus autosampler (Jasco International Co., Ltd., Tokyo, Japan) and a CO-2060 Plus column oven (Jasco International Co., Ltd., Tokyo, Japan). Detection was carried out with an UV-2070 Plus visible detector (Jasco International Co., Ltd., Tokyo, Japan). The data were processed with ChromNAV (software version 2.3). The mobile phases were A, acetic acid in bi-distilled water at 2% and B, acetonitrile and acetic acid in bi-distilled water (30%, 2%, respectively) at constant flow rate of 1 mL/min. All solutions were filtered through a 0.22 μm membrane filter. All eluents were HPLC grade and the applied elution gradient is shown in Table 1.

The injected volume was 20 µL, and the detection of peaks was performed at 280 nm.

Stock solutions of 1 mg/mL of each standard were prepared. Seven working standard solutions were prepared at concentrations of 500, 250, 125, 62,5, 31,25, 15,625 and 7.8125 ug/mL by dilution in the B mobile phase from the stock solutions.

### 2.7. Statistical Analysis

The results are the means ± SD (standard deviation) of three independent experiments. The significance of differences among means was determined by one-way ANOVA (CoStat, Cohort 6 software). The comparisons among means were performed by Tukey’s test (*p* < 0.05).

## 3. Results and Discussion

In this work, we focused our attention on strains showing growth in the presence of OMWW at the same time; hence, on strains that putatively might use this agri-food wastewater as a potential growth medium to implement the population biomass; and on the its ability to remove phenolic compounds. The five treated microalgae responded very differently to the vegetation water samples, especially when different dilutions were used. In fact, some microalgae have shown a greater aptitude towards tolerating higher concentrations of OMWW and others have shown greater aptitude towards lower concentrations.

**Figure 1:**
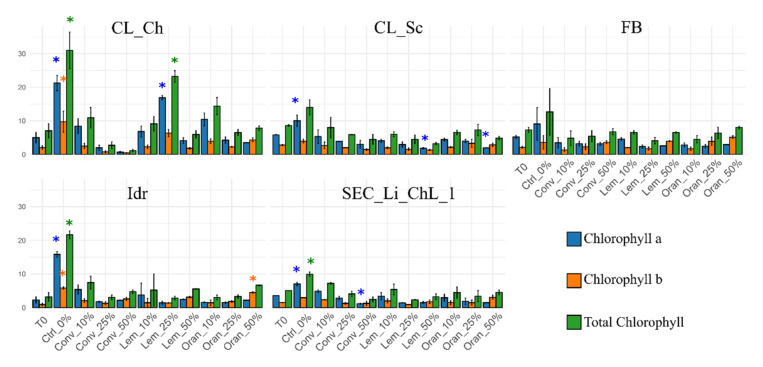
Pigment contents of the first screening experiment; asterisks indicate statistically significant differences between tests and the pigment content of T0 (the microalgal culture at the beginning of the experiment). “Conv” samples: vegetation water obtained with traditional method; “Lem” samples: vegetation water obtained with lemon peels extraction; “Oran” samples: vegetation water obtained with orange peels extraction. Ctrl_0% samples: only TAP medium.

In both our experiments, all of the *Chlorella* sp. microalgal strains were able to grow in the experimental conditions in the absence of vegetation water for the whole experimental duration: accordingly, an increment in the total chlorophyll content was measured for all five microalgal strains after 1 week (i.e., T0 values vs. Ctrl_0%, Figure 1).

The 7-day experimental duration was chosen on the basis of previously conducted experiments with the same algal species [16,18] and on the basis of similar experiments testing microalgae in the presence of OMWW (i.e., 10-day duration) [22,23].

The measurement of photosynthetic pigment content revealed that a one-week treatment induced an increase in the total chlorophyll content in Idr strains in 6 out of 10 treatments (Ctrl_0%, Conv_10%, Conv_50%, Lem_10%, Lem_50% and Oran_50%). Interestingly, its tolerated high concentrations of vegetative water based on three olive-oil extraction methods (Conv_50%, Lem_50% and Oran_50%). An increment in chlorophyll content was also observed in Cl_Ch (under the treatments Conv_10%, Lem_10% and 25% and Oran_10%) and SEC_Li_ChL_1 exposed to Conv_10% vegetation water. On the contrary, a decrease in the investigated pigments was noted in CL_Sc and FB strains in almost all different VWs and concentrations with some exceptions concerning chlorophyll b (Figure 1). Noteworthy is the intolerance of CL_Ch strains exposed to Conv 25% and 50%. In fact, a loss of about 20–35% was observed in the photosynthetic pigment content in comparison with time T0 even though it was the one showing the greatest increment especially in the presence of the lemon 25% treatment. Interestingly, in some cases, we observed higher values of chlorophyll b than chlorophyll a, especially in strains exposed to the highest OMWW concentrations; these are the cases of CL_Ch and CL_Sc exposed to Oran_50% treatment; of the FB strain exposed to Conv_50%, Lem_50%, Oran_25% and Oran_25% treatments; of the Idr strain in presence of Conv_50%, Oran_25% and Oran_25%; and of strain SEC_Li_ChL_1 with Lem and Oran 50% treatments. This observation might be explained if we consider that the highest concentrations of vegetation water might represent a stressful condition for our strains; indeed, according to the literature, stress might induce an increased production of chlorophyll b with respect to chlorophyll a and, consequently, a decrease in the chlorophyll a/b ratio [24,25]. Moreover, changes in pigment contents in microalgae can be considered an adaptation mechanism in high light conditions, and the ratio of Chl a/b as well as Carotenoid/total Chl increase with an increase in light intensity [26]. Considering that the vegetation water has a dark purple color at all of the tested concentrations, our strains were in a situation of light scarcity, and this might have caused a decrease in the chlorophyll a/b ratio.

Regarding the phenol reduction, the results are shown in Table 2; decreases of 21.8% and 26.5% in all microalgal strains growing in OMWW obtained from pressing with orange and lemon at lower concentrations (10%), respectively, were observed. The Idr strain pointed out the greatest tolerance in this low concentration, especially in the presence of lemon with a percentage of reduction of 53.9%. This phenol reduction reflects an increment in pigment content with respect to the beginning of the experiment (T0), suggesting not only decontamination activity of such a strain but also growth in terms of biomass. Contradictory to the growth increment (Figure 1), strain CL_Sc is apparently more predisposed toward tolerating higher concentrations (25 and 50%) in both Lem and Conv treatments, which translated into its better efficacy in phenolic reductions (28.2% and 30.3%, respectively). In light of such data, we can hypothesize that, despite a reduced growth of this strain in the presence of 25% and 50% lemon and control treatments, the microalgal strain is much more effective in reducing phenols. A similar behavior was observed also in SEC_LI_ChL_1 exposed to Conv_25%, with a reduction in phenolic compounds of 21%, and in FB strains treated with Conv and Lem at 10% (with percent reductions of 32.4% and 33.5%, respectively).

**Figure 2:**
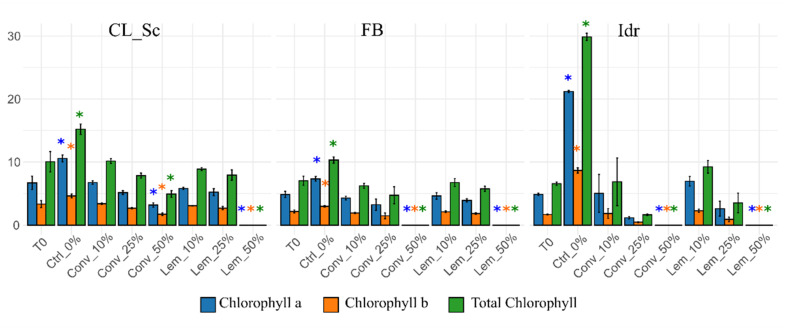
Pigment contents of the second experiment; asterisks indicate statistically significant differences between tests and the pigment content of T0 (the microalgal culture at the beginning of the experiment). “Conv” samples: vegetation water obtained with traditional method; “Lem” samples: vegetation water obtained with lemon peels extraction. Ctrl_0% samples: only TAP medium.

Considering the combined results obtained on the first experiment, related to both the growth of the strains and their capacity in reducing phenolic compounds, the second experiment was performed on only three microalgal strains (Idr, CL_Sc and FB) and on the vegetation water from conventional extraction and extraction with lemon methods. The results related to the microalgal growth (Figure 2) were in line with the preliminary experiment showing that all three strains were able to survive in the presence of vegetation water from conventional (Conv) and from lemon (Lem) methods up to a concentration of 25%. These results obtained from both the first and the second experiments demonstrate a higher tolerance of greater concentrations of OMWW of the selected strains with respect to the *Chlorella vulgaris* strain, which were able to grow up to 6% *v/v* OMWW [23]. The OMWWs used might represent three different culture media, with different potentials for adaptation; in particular, the orange OMWW was the least effective. On the contrary, the lemon and control OMWWs represent excellent candidates for an industrial application. Further investigations in this direction might be addressed in the future.

Analyzing the percentage of reduction in phenolic compounds (Table 3), it is possible to observe that strain CL_Sc tolerated high concentrations of OMWW very well, showing a reduction in phenolic compounds of 52.9% with the traditional vegetation water treatment, while the Idr strain tolerated low concentrations and evidenced a reduction of approximately 45% in both tested samples (conventional and lemon), with a better yield at low concentrations (Table 3). On the contrary, FB strain worked better at intermediate OMWW concentrations in both tested samples (Conv and Lem), and the best results were obtained with the lemon OMWW sample with a reduction of 50.3%.

All of these data revealed the greatest ability of our *Chlorella* sp. strains in removing phenolic compounds from OMWWs with respect to similar work testing the same microalgal genera, where only 12% were removed under light condition [23]. Indeed, according to the recent literature, *Chlorella vulgaris* was able to remove about 12% of phenolic compounds after 9–10 days of cultivation in light conditions [23]. Other microalgal strains recently tested for the same purposes revealed a greater ability to remove phenols from OMWW, namely *Acutodesmus obliquus* and *Monoraphidium braunii* (21 and 17% of phenolic compounds removed, respectively, in 9–10 days during experiments in light conditions [23]). Furthermore, our results highlight the greater removal ability of our isolated environmental strains in OMWW treatments, reaching removal percentages greater than 50% in certain cases.

Interestingly, other than obtaining a “decontaminated” WW, microalgal application in OMWW remediation might also produce an “enriched” biomass since phenolic compounds might be accumulated within the microalgal cells. Once the biomass is recovered and characterized from a nutraceutical viewpoint, it might represent the starting point for the investigations on its reuse as feed/food supplements, under a circular economy context. This aspect might open new perspectives for the development of a new agri-food chain derived from food waste, maybe focused on the reuse of the “enriched” biomass for feed/food purposes [27]. This circular process, other than reducing the costs for the remediation of a contaminated matrix from a food industry process, might also open new opportunities for the recovery of the biomass and, thus, new opportunities from an economical viewpoint.

In this scenario, it is anyway mandatory to consider two important aspects: (i) the OMWW is not a sterile matrix, and it possibly harbors a bacterial/fungal community; (ii) microalgae are surrounded by microorganisms composed of their phycosphere, with important roles in all of the microalgal population growth/survival [28]. Hence, we cannot a priori exclude that these microorganisms might have an additional effect in phenol transformations. On the other hand, the possibility to sterilize the OMWW (i.e., by autoclaving) might affect the original phenol composition, and it is not a practical operation in industrial production; moreover, the microorganisms of the phycosphere are in most cases not removable under laboratory conditions, since they are closely related to microalgal cells and have a role in their survival [29].

### HPLC Analysis of Phenolic Compounds

Hydroxytyrosol and tyrosol were the main phenolic compounds present in the OMWW methanolic extract (Table 4), accounting for 2.1 mg and 0.4 mg, respectively. These two phenols were also of high value in both lemon and orange samples. Interestingly, between these samples, only a slight variation was observed in tyrosol content while that of hydroxytyrosol was halved in the lemon sample but then increased to almost 10% in the orange sample in comparison with the control.

Both the orange and lemon samples underlined the presence of coumaric acid, which was mostly represented by 4-coumaric acid in the case of orange (0.03 mg) and by 4-coumaric acid with 2-coumaric acid in lemon samples (0.06 mg and 0.04 mg, respectively). The complete removal of tyrosol and hydroxytyrosol was observed in the FB strain cultivated at a high rate of 25% (*v*/*v*) OMW and in CL_Sc, grown at 10% OMW and lemon as well as at 25% lemon. On the contrary, Idr was not able to reduce the content of, especially, tyrosol. These data agreed with that in the literature since the degradation of coumaric acid, tyrosol and hydroxytyrosol have already been described by means of the microalgae *Ankistrodesmus braunii* and *Scenedesmus quadricauda* [21,30].

According to the recent literature, microalgae belonging to the *Scenedesmus* sp. genus not only are able to grow on OMWW and remove sugars and phenols [31] but also accumulate polyphenols successfully removed from the WW inside their cells [32]. Hence, we cannot exclude the possibility that the *Chlorella* sp. strains tested in this work might also accumulate intracellular levels of the different phenolic compounds removed from the OMWW (Table 4). If so, the potential application of their harvested biomass as feed/food supplements might be of interest for future investigations.

## 4. Conclusions

This experiment, tested on five different *Chlorella sorokiniana* microalgal strains of environmental origin, allowed us to verify the possibility of adaptation to OMWW and brought about good results for possible use at an industrial level, with the aim of reducing pollution from the olive-oil supply chain and with the intent to generate by-products that can be used in different chains for the production of cosmetics and food supplements.

In conclusion, strains CL-Sc, Idr and FB from Lake Massaciuccoli showed high potential in reducing total phenolic compounds from the OMWW, opening new perspectives for the biological treatment of such wastewater.

## Figures and Tables

**Table 1:** The elution gradient for the HPLC determinations.

T (min)	A%	B%
0	90	10
25	70	30
55	5	95
60	90	10

**Table 2:** Total phenols at the starting time and their percentage reduction after one-week treatments in the preliminary screening performed on all five microalgal strains (only the best data are reported).

	OMWW Sample	Total Phenols at the Starting Time (g/L Gallic Acid)	% Reduction $\left[\left(\left(C_{0}-C_{f}\right)/C_{0}\right)\times 100\right]$
**SEC_LI_ChL_1**	10% CONTROL	0.39 ± 0.05 f	5.3 ± 1.1 h
	10% ORANGE	0.58 ± 0.02 e	1.3 ± 1.0 i
	10% LEMON	0.35 ± 0.01 f	2.5 ± 1.3 i
	25% CONTROL	0.82 ± 0.05 c	21.0 ± 1.1 e
**CL_Sc**	10% CONTROL	0.39 ± 0.04 f	18.7 ± 1.0 f
	10% ORANGE	0.58 ± 0.02 e	9.9 ± 1.0 g
	10% LEMON	0.35 ± 0.01 f	24.0 ± 1.3 d
	25% CONTROL	0.82 ± 0.06 c	27.0 ± 1.0 d
	25% LEMON	0.73 ± 0.03 d	28.2 ± 1.0 d
	50% CONTROL	1.54 ± 0.05 a	30.3 ± 1.2 c
**CL_Ch**	10% CONTROL	0.39 ± 0.03 f	13.9 ± 1.0 g
	10% ORANGE	0.58 ± 0.02 e	21.8 ± 1.1 e
	10% LEMON	0.35 ± 0.06 f	26.5 ± 1.1 d
	25% ORANGE	1.31 ± 0.03 b	11.8 ± 1.0 g
	25% LEMON	0.73 ± 0.02 d	10.4 ± 1.0 g
	50% LEMON	1.36 ± 0.04 b	4.0 ± 1.2 h
**Idr**	10% CONTROL	0.39 ± 0.02 f	45.8 ± 1.0 b
	10% LEMON	0.58 ± 0.01 e	53.9 ± 1.3 a
**FB**	10% CONTROL	0.39 ± 0.06 f	32.4 ± 1.0 c
	10% ORANGE	0.58 ± 0.03 e	16.5 ± 1.3 f
	10% LEMON	0.35 ± 0.02 f	33.5 ± 1.2 c
	25% CONTROL	0.82 ± 0.01 c	17.5 ± 1.0 d
	25% LEMON	0.73 ± 0.04 d	18.4 ± 1.1 f

Data are expressed as mean ± SD; in the same column, the letters (a–i) indicate significant differences (*p* < 0.05) after the analysis of variance (ANOVA).

**Table 3:** Total phenols at the starting time and their percentage reduction after one-week experiment in the second experiment performed on the best microalgae strains (only the best data are reported).

	OMWW Sample	Total Phenols at the Starting Time (g/L Gallic Acid)	% Reduction $\left[\left(\left(C_{0}-C_{f}\right)/C_{0}\right)\times 100\right]$
**CL_Sc**	10% CONVENTIONAL	0.39 ± 0.03 d	24.4 ± 1.0 d
	10% LEMON	0.35 ± 0.04 d	35.3 ± 1.1 c
	25% CONVENTIONAL	0.82 ± 0.01 b	32.0 ± 1.1 c
	25% LEMON	0.73 ± 0.05 c	34.9 ± 1.0 c
	50% CONVENTIONAL	1.54 ± 0.06 a	52.9 ± 1.0 a
**Idr**	10% CONVENTIONAL	0.39 ± 0.02 d	45.7 ± 1.3 b
	10% LEMON	0.35 ± 0.04 d	44.8 ± 1.0 b
**FB**	10% CONVENTIONAL	0.39 ± 0.03 d	26.7 ± 1.0 d
	10% LEMON	0.35 ± 0.02 d	34.0 ± 1.3 c
	25% CONVENTIONAL	0.82 ± 0.03 b	45.1 ± 1.2 b
	25% LEMON	0.73 ± 0.05 c	50.3 ± 1.0 a

Data are expressed as mean ± SD; in the same column, the letters (a–d) indicate significant differences (*p* < 0.05) after the analysis of variance (ANOVA).

**Table 4:** Amount (mg) of identified phenolic compounds in the control (t = 0) and CL-Sc, Idr and FB cultivation at 10%, 25% and 50%.

	OMWW Sample	Tyrosol	Hydroxityrosol	4-Coumaric Acid	2-Coumaric Acid
**T = 0**	CONVENTIONAL	0.40	2.10	-	-
	LEMON	0.30	1.00	0.06	0.04
	ORANGE	0.50	2.30	0.03	-
**CL-Sc**	10% CONVENTIONAL	-	-	-	-
	10% LEMON	-	-	-	-
	25% CONVENTIONAL	0.01	0.01	-	-
	25% LEMON	-	-	-	-
	50% CONVENTIONAL	0.03	0.05	-	-
**Idr**	10% CONVENTIONAL	0.04	-	-	-
	10% LEMON	0.03	-	-	-
**FB**	10% CONVENTIONAL	0.06	-	-	-
	10% LEMON	0.01	-	-	-
	25% CONVENTIONAL	-	-	-	-
	25% LEMON	0.03	-	-	-

## Data Availability

Data is contained within the article.

## Supplementary Materials

The following supporting information can be downloaded at https://www.mdpi.com/article/10.3390/foods11101398/s1, Figure S1: Emission spectra of the pellets and supernatants of the three different vegetation waters: (a) control, (b) lemon and (c) orange. Bars represent the standard error calculated on three replicate spectrophotometric measures.
