# Supplementary material for: Application of Five Different Chlorella sp. Microalgal Strains for the Treatment of Vegetation Waters Derived from Unconventional Oil Extractions Enriched with Citrus Byproducts
Source: Foods. 2022 May 12;11(10):1398. doi: 10.3390/foods11101398 (PMC9141023; doi:10.3390/foods11101398)
Supplement: Supplementary file 1 [file foods-11-01398-s001.zip › foods-1700541-supplementary.pdf]

Supplementary Material

Application of Five Different *Chlorella* sp. Microalgal Strains for the Treatment of Vegetation Waters Derived from Unconventional Oil Extractions Enriched with Citrus Byproducts

Monica Macaluso[1§], Carolina Chiellini[2§*], Adriana Ciurli[1], Lorenzo Guglielminetti[1,3], Basma Najar[1], Isabella Taglieri[1], Chiara Sanmartin[1], Alessandro Bianchi[1], Francesca Venturi[1,3] and Angela Zinnai[1,3]

[1] Department of Agriculture, food and Environment, University of Pisa, Via del Borghetto 80, 54126 Pisa (Italy)
[2] Italian National Research Council, Institute of Agricultural Biology and Biotechnology Via Moruzzi, 1 56124 Pisa (Italy)
[3] Interdepartmental Research Centre "Nutraceuticals and Food for Health", University of Pisa, Via del Borghetto 80, Pisa, 56124, Italy
§ these authors contributed equally to the work
* Correspondence: author:
Carolina Chiellini: carolina.chiellini@ibba.cnr.it.

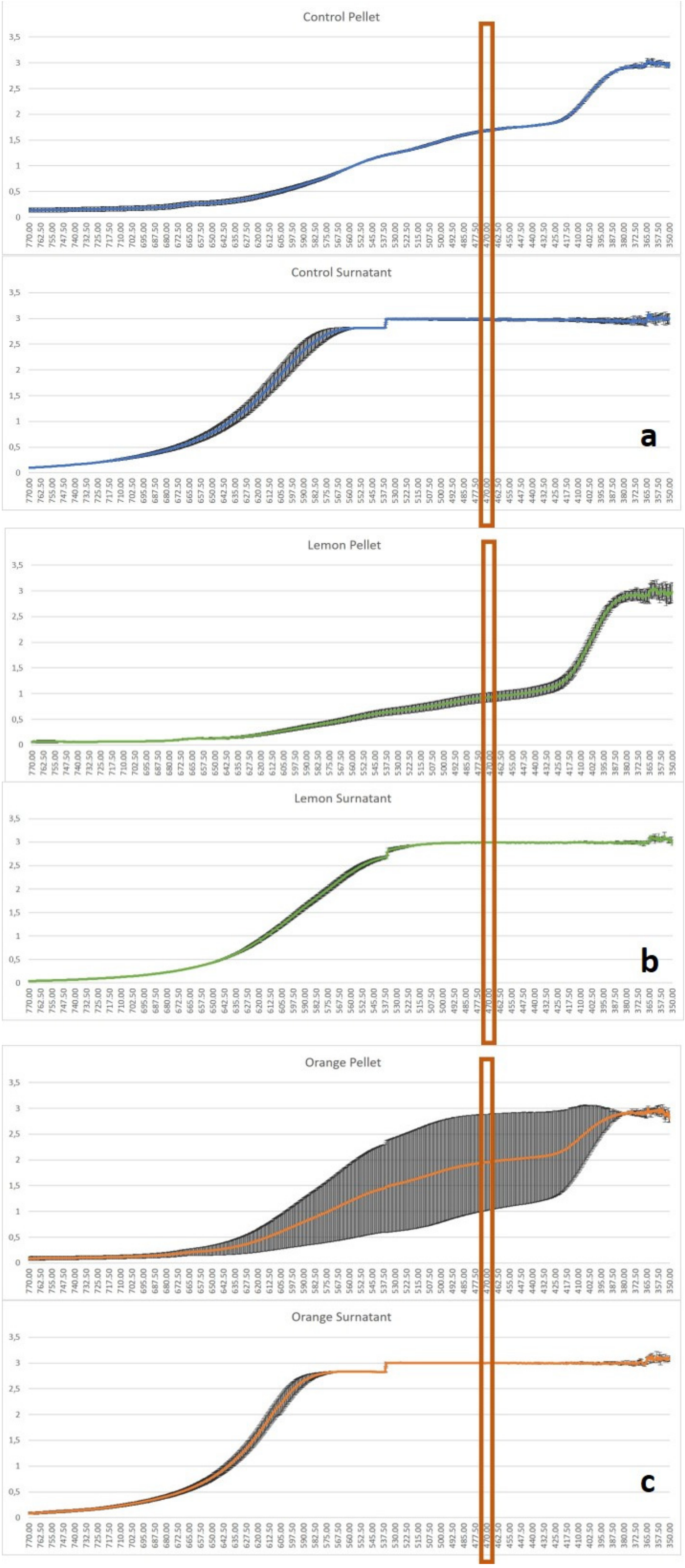

Supplementary Figure S1. Emission spectra of the pellets and supernatants of the three different vegetation waters: (**a**) control, (**b**) lemon and (**c**) orange. Bars represent the standard error calculated on three replicate spectrophotometric measures.
